# Acquired Refractory Angioneurotic Edema in a Known Case of Systemic Lupus Erythematosus

**DOI:** 10.7759/cureus.31382

**Published:** 2022-11-11

**Authors:** Shubham V Nimkar, Pallavi Yelne, Shilpa A Gaidhane, Sourya Acharya, Sunil Kumar, Keyur Saboo

**Affiliations:** 1 Department of Medicine, Jawaharlal Nehru Medical College, Datta Meghe Institute of Medical Sciences, Wardha, IND; 2 School of Epidemiology and Public Health, Jawaharlal Nehru Medical College, Datta Meghe Institute of Medical Sciences, Wardha, IND

**Keywords:** c1 inhibitor, steriods, angioneurotic, sle, acquired

## Abstract

A woman came to the emergency room with swelling of the face, which included swelling around the lips and the eyes. The patient had undergone root canal treatment under lidocaine anesthesia one day prior, after which she developed swelling. Because angioneurotic edema was a possibility, the complement components C3 and C4 and C1 esterase inhibitor (C1-INH) were tested. The C4 level was found to be very low (0.08 gm/l) and the C1 level was also on the lower side (0.26 gm/l). Angioneurotic edema with acquired C1-INH deficiency was diagnosed after complete systemic and physical examinations. The patient made a complete recovery with the help of steroids, fresh frozen plasma, antibiotics, and antiallergic medications. For its rarity, this case of systemic lupus erythematosus refractory angioneurotic edema with acquired C1-INH deficiency is being reported.

## Introduction

Acquired angioedema (AAE) is a rare disorder that causes recurring episodes of non-pitting edema around the lips, eyes, upper and lower limbs, and genitalia. When this swelling lasts for several days, it is called resistant angioedema. When swelling involves the tongue and larynx, it becomes a life-threatening condition due to upper-airway obstruction. AAE can also cause edema of the intestines and alveoli, which can be fatal. Mostly, angioedema is a mild and self-limiting disease, but it may cause death in 15% to 30% of cases [1].

Angioedema is caused by the absence or decreased level of C1 esterase inhibitor (C1-INH) enzyme. It can be hereditary or acquired. C1-INH inhibits proteins like kallikrein and coagulation factor 12, which are involved in inflammation and coagulation. C1 INH deficiency causes unregulated activation of the classical complement pathway.

The first symptom of AAE in lymphoma patients, monoclonal gammopathy of undetermined significance (MGUS) emerged in 1971 [2]. There are many potential causes of swelling episodes, including pregnancy, specific meals, moderate trauma (like dental work), viral illnesses, cold exposure, and emotional stress. Angioedema in lupus patients may be brought on by an acquired form of C1-INH deficiency, most likely as a result of the development of antibodies against the C1-INH molecule. If one is not aware of the various manifestations of systemic lupus erythematosus, it might be misdiagnosed or overlooked [3].

Angioedema is one of those unusual manifestations for which the diagnosis of lupus is readily neglected. Patients with systemic lupus erythematosus (SLE) have a greater rate of adverse medication responses, according to the literature [4]. A cross-sectional study conducted by Yiming Luo et al. demonstrated that SLE is associated with higher odds of having angioedema, including severe angioedema as the principal reason for inpatient admission. SLE is possibly an independent risk factor for angioedema [5].

## Case presentation

**Figure 1:**
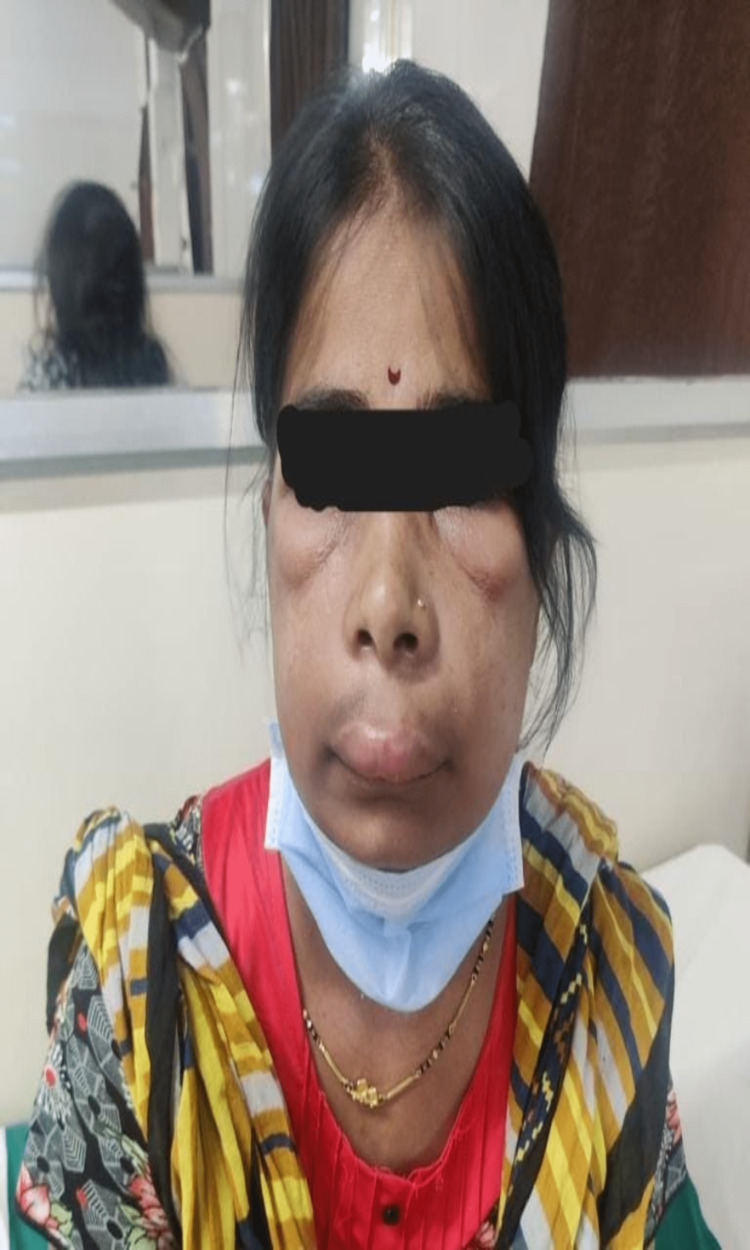
Suggestive of swelling over upper eyelids and infraorbital region

**Figure 2:**
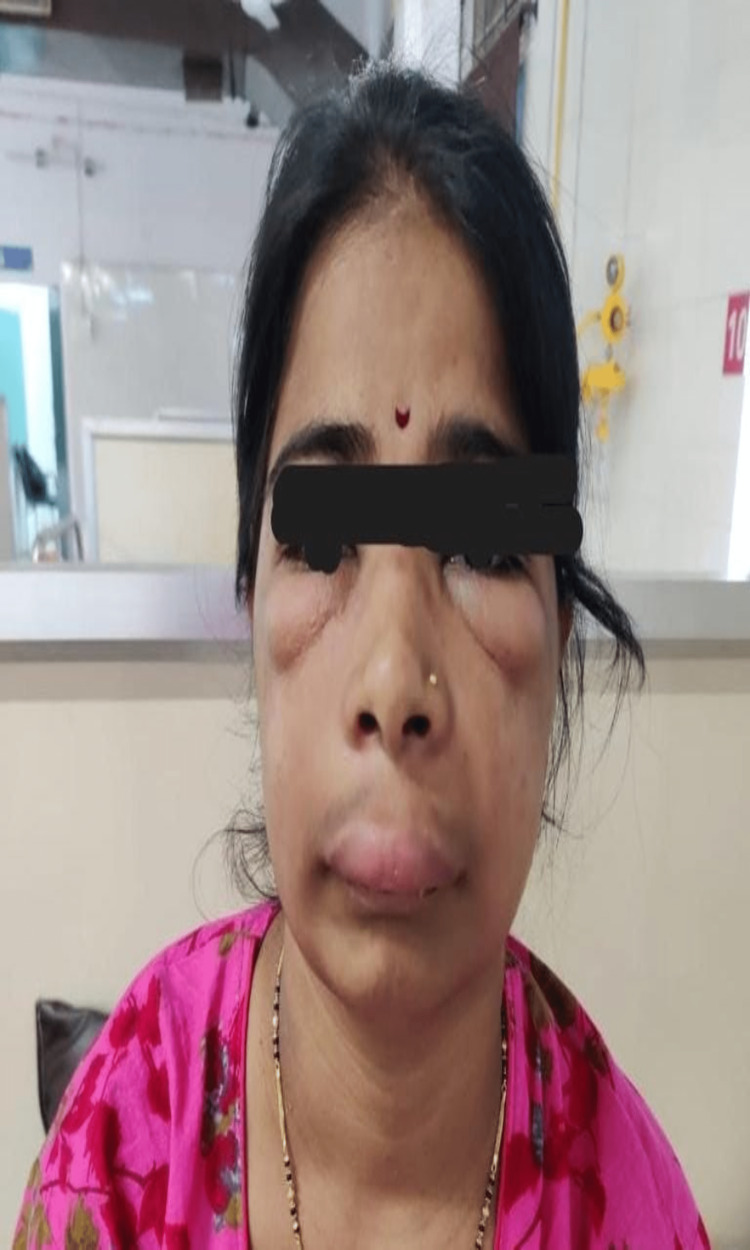
Suggestive of swelling over upper eyelids and infraorbital region

A 38-year-old female patient presented with swelling of recent origin, which was developed within half an hour over the upper lip, and infraorbital region (Figure 1, 2). She was diagnosed with SLE three years ago. She was being treated with 200 mg of hydroxychloroquine, folic acid, 25 mg of methotrexate, and prednisolone. She presented to the dental outpatient department for root canal treatment. Local anesthesia with lignocaine was administered. The patient underwent root canal treatment successfully and without any complications. After 30 minutes of the procedure, the patient developed facial edema. The patient was brought to the medicine outpatient department by the dental resident. She had no previous history of similar attacks when she had previously received lignocaine during her delivery and no family history of angioedema. Upon general physical examination, the patient was moderately built and afebrile, with a pulse rate of 90/min, respiratory rate of 18/min, blood pressure of 130/90 mmHg, and no elevated jugular venous pressure (JVP). The patient had tender erythematous swelling over the infraorbital region upper lip. The patient has no comorbidities like hypertension, diabetes mellitus, asthma, or tuberculosis. Lymph nodes were not palpable. Other than the face, the swelling was not noted on any other region.

Complete blood count investigations on cell count with peripheral smear showed 11.1 g/dL hemoglobin, 32.5 g/dl mean corpuscular hemoglobin concentration, 72.2 p mean corpuscular volume, and 23.5 pg mean corpuscular hemoglobin. Total red blood cell count was 4.71 million/mm3, white blood cell count was 10100/ microlitre, and platelet count was 3.10/microlitre of blood. Prothrombin time was 12.5 seconds, activated partial thromboplastin clotting time was 30.20 seconds, and the international normalized ratio was 1.28. Random blood sugar was 151 mg/dL. Kidney function test showed urea: 18 mg/dl, creatinine: 0.6 mg/dl, serum sodium: 147 mmol/L, and serum potassium: 4.4 mmol/L. Liver function test showed serum albumin: 4.8 g/dl, alkaline phosphatase level was 100 IU/l, bilirubin conjugated (0.2 mg/dL), bilirubin unconjugated (0.1 mg/dL), globulin (3.6 g/dL), serum glutamic pyruvic transaminase (15 U/L), serum glutamic oxaloacetic transaminase (30 U/L), total protein (8.4 g/dL), and total bilirubin (0.3mg/dL). Complement 3 (C3): 0.81 gm/l, complement 4 (C4): 0.08 gm/l, C1-INH: 0.26 gm/l, and 24-hour urinary protein: 1220 mg/dl. Blood tests and diagnostic workup of the patient are provided in Table 1.

**Table 1:** Blood Tests

Blood Tests and Diagnostic Workup	Result	Reference Range
Hemoglobin	10.7 gm%	12-15 gm%
Mean corpuscular hemoglobin concentration	32.5%	31.5-34.5%
Mean corpuscular volume	72.2fl	80-100 fL
Mean corpuscular hemoglobin	23.5 pico-gm	27-32 pico-gm
Total red blood cell count	4.71 millions/cu.mm	3.8-4.8 millions\cu.mm
Total white blood cell count	10100/cu.mm	4000-10000 cu.mm
Platelet count	3.10 Lacs/cu.mm	1.50-4.10Lacs/cu.mm
Random blood sugar	151 mg%	30-150mg%
Urea	18 mg/dl	15-36 mg/dl
Creatinine	0.6 mg/dl	0.52-1.04mg/dl
Serum sodium	147 mmol/L	137-147 mmol/L
Serum potassium	4.4 mmol/L	3.3-5.1 mmol/L
Serum albumin	4.8 g/dl	3.5-5.0g/dl
Alkaline phosphatase	100 U/l	38-126U/l
Bilirubin conjugated	0.2 mg/dL	0.0-0.3 mg/dl
Bilirubin unconjugated	0.1 mg/dL	0.0-1.1 mg/dl
Globulin	3.6 g/dL	2.4-3.5gm/dl
Serum glutamic pyruvic transaminase (SGPT)	15 U/L	<35 U/l
Serum glutamic-oxaloacetic transaminase (SGOT)	30 U/L	14-36 U/l
Total protein	8.4 g/dL	6.3-8.2 g/dl
Total bilirubin	0.3mg/dL	0.2-1.3 mg/dl
Complement 3 (C3)	0.81 gm/l,	0.88-2.01 gm/l
Complement 4 (C4)	0.08 gm/l	0.15-0.45 gm/l
C1 esterase inhibitor	0.26 gm/l	0.16-0.33gm/l

The patient developed post-pregnancy SLE three years ago. So the diagnosis of lignocaine-induced acquired angioedema was made and the patient was started with injectable steroids injection hydrocortisone 100mg IV thrice daily and injection pheniramine 2cc IV thrice daily with other supportive management. After 48 hours with no expected improvement in the patient, a diagnosis of refractory AAE in SLE was made. The patient was given a transfusion of four units of fresh frozen plasma. As fresh frozen plasma contains C1 esterase enzyme, fresh frozen plasma was transfused.

After starting treatment with an injection of tranexamic acid, fresh frozen plasma transfusion, and antiallergic treatment, the swelling started subsiding from day three, and the swelling completely resolved on day six. The patient’s condition improved throughout the hospital stay. The patient was discharged with stable vitals on day six and was doing well on follow-up.

## Discussion

Angioedema can be caused by kinin or mast cells, with mast cell-mediated angioedema being more prevalent. Further classification divides kinin-mediated angioedema into two categories: acquired and inherited [6]. While Type 2 hereditary angioedema has normal levels of C1-INH but is functionally deficient, Type 1 hereditary angioedema has inherently low levels. Comparatively speaking, AAE is less common than hereditary angioedema (HAE) [7]. While Type 2 AAE might have normal or increased levels of functionally inactive C1-INH, Type 1 AAE has lower serum levels of functionally active C1 INH. Type 1 AAE happens when C1-INH is consumed excessively, as in B cell lymphoproliferative neoplasm, and Type 2 AAE happens when C1-INH autoantibodies are present [8].

The primary cause of our patient's AAE was SLE. AAE can be diagnosed in the absence of any past history of comparable complaints or any family history of angioedema. Our patient exhibited very low levels of C4, which indicated complement consumption, and lower levels of C1, which met the AAE Type 1 profile [9]. SLE is the most common autoimmune disease (AD) co-occurring with HAE Type I and II. Cause and effect for co-occurring HAE and AD have not been clinically established but could be related to a lack of sufficient C1-INH function. SLE can also present as facial necrotizing fasciitis, pancreatic pseudocyst, and haemophagocytic syndrome [10-12].

Management

The usage of C1-INH pure concentrate in therapy is well established [13]. Due to the intensified catabolism of C1-INH by auto-antibodies, substantial dosages are required to treat AAE, roughly 12,000 U, which is significantly higher than the amount advised for HAE (500-1,500 U) [6,13]. In the rural setup of India, it is not readily available.

Another option is to provide fresh frozen plasma, but because it has high amounts of complements, it may make the prior symptomatology worse, [6,14] and hence patients should be monitored. For long-term prophylaxis, antifibrinolytics such as tranexamic acid should be administered. In HAE, it is widely known that attenuated androgens such as danazol and stanozolol are used to promote the liver's production of C1-INH. Recently, it has been demonstrated that the use of rituximab (chimeric anti-CD20 antibody) in the treatment of AAE is successful in attaining remission of the episode of angioedema in instances that are nonresponsive and challenging to control [15-18]. Few studies have shown the efficacy of corticosteroids and plasmapheresis in conjunction with immunosuppressants (azathioprine, cyclosporin) [19].

## Conclusions

In this case, the SLE refractory angioedema with acquired C1-INH deficiency was aggressively treated at first with injectable steroids and then with fresh frozen plasma and other supportive treatments, which resolved the symptoms of the patient. In patients of SLE refractory angioedema with acquired C1-INH deficiency, it is suggested to treat them aggressively with injectable steroids, fresh frozen plasma, and other supportive treatments for resolving symptoms and betterment of the patient. 
